# Fault-tolerant pedigree reconstruction from pairwise kinship relations

**DOI:** 10.1093/bioinformatics/btag251

**Published:** 2026-05-04

**Authors:** Edward C Huang, Kevin A Li, Vagheesh M Narasimhan

**Affiliations:** Department of Computer Science, Stanford University, Stanford, CA 94305, United States; Department of Archaeogenetics, Max Planck Institute for Evolutionary Anthropology, Leipzig, Saxony 04103, Germany; Department of Computer Science, The University of Texas at Austin, Austin, TX 78712, United States; Department of Integrative Biology, The University of Texas at Austin, Austin, TX 78712, United States; Department of Statistics and Data Sciences, The University of Texas at Austin, Austin, TX 78712, United States; Oden Institute for Computational Engineering and Sciences, The University of Texas at Austin, Austin, TX 78712, United States

## Abstract

**Motivation:**

Pedigrees reconstructed from biologically related ancient genomes have revealed many insights into (pre)history. To our knowledge, all reported ancient pedigrees have been primarily manually reconstructed, as existing pedigree reconstruction methods are ill-suited for the quality and nature of ancient DNA data.

**Results:**

We introduce repare, an open-source software method to automatically reconstruct pedigrees from inferred pairwise kinship relations, which are readily obtainable from ancient genomes. This method reconstructs pedigrees by iteratively incorporating pairwise kinship relations into a set of candidate pedigrees, with pruning and sampling to reduce its search space. It optionally considers supporting information such as haplogroups and skeletal age-at-death estimates. We evaluate this method on a variety of simulated pedigrees with varying error rates and missingness. We also use this method to reconstruct several published pedigrees that were originally manually reconstructed; for one, we present a potential alternative topology. repare optionally incorporates user-inferred pedigree constraints, enabling “human-in-the-loop” reconstruction workflows. Especially when used with these user-inferred constraints, we find that repare represents a powerful and flexible tool for ancient pedigree reconstruction.

**Availability and implementation:**

repare is freely available at https://github.com/Narasimhan-Lab/repare. In addition, source code, benchmark scripts, and benchmark results used in this work are archived at https://doi.org/10.5281/zenodo.19716772.

## 1 Introduction

Advances in ancient DNA (aDNA) sequencing methods have enabled the sampling of ancient genomes from multiple individuals interred at the same archaeological site. Notably, a set of biologically related ancient genomes can be arranged into a pedigree, which in turn allows for analysis of inheritance patterns, population structure, and other social and biological trends through time. There exist many pedigree reconstruction methods built primarily for modern genetic samples (Riester *et al.* 2009, Cussens *et al.* 2013, Shem-Tov and Halperin 2014, Staples *et al.* 2014, He *et al.* 2017, Huisman 2017, Ko and Nielsen 2017, Jewett *et al.* 2021); these methods have seen little, if any, usage with ancient datasets. This is at least partially due to the unique nature of aDNA data: calls are often “pseudo-haploid” due to low sequence coverage (Mallick *et al.* 2024), contamination and damage rates are elevated (Ginolhac *et al.* 2011), exact birth years are unknowable with current dating techniques (Sedig *et al.* 2021), and pedigrees are rarely completely sampled and can span many generations. These limitations break many of the assumptions of existing pedigree reconstruction methods and make it exceedingly difficult to reconstruct ancient pedigrees using methods not purpose-built for aDNA data.

As an example, PRIMUS is a state-of-the-art pedigree reconstruction method (designed for modern pedigrees) that utilizes identity-by-descent (IBD) proportion estimates (Staples *et al.* 2014). However, PRIMUS is not designed to reconstruct consanguineous pedigrees, which can be common in ancient contexts. In addition, accurate IBD inference in ancient samples requires at least 1.0× mean coverage on the 1240k single nucleotide polymorphism capture panel (Ringbauer *et al.* 2024), which precludes the inclusion of many ancient samples. For example, the Hazleton North site has seven individuals with <1.0× mean 1240k coverage out of 35 reported individuals (Fowler *et al.* 2022), and the Gurgy “les Noisats” site has 35 individuals with <1.0× mean 1240k coverage out of 94 reported individuals (Rivollat *et al.* 2023). For these reasons, to our knowledge, all reported ancient pedigrees have been primarily manually reconstructed (Mittnik *et al.* 2019, Schroeder *et al.* 2019, Fowler *et al.* 2022, Blöcher *et al.* 2023, Chyleński *et al.* 2023, Rivollat *et al.* 2023, Gnecchi-Ruscone *et al.* 2024, Penske *et al.* 2024, Rodríguez-Varela *et al.* 2024, Seersholm *et al.* 2024, Szécsényi-Nagy *et al.* 2025, Wang *et al.* 2025a, 2025b, Yüncü *et al.* 2025).

Despite aDNA’s relatively lower quality, it is feasible to infer pairwise kinship relations from ancient samples with as low as 0.05× average sequence coverage, albeit at non-negligible error rates (Lipatov *et al.* 2015, Kuhn *et al.* 2018, Hanghøj *et al.* 2019, Waples *et al.* 2019, Fernandes *et al.* 2021, Nyerki *et al.* 2023, Popli *et al.* 2023, Alaçamlı *et al.* 2024, Lefeuvre *et al.* 2024, Rohrlach *et al.* 2025). As such, many ancient pedigrees have been manually reconstructed from inferred kinship relations along with supporting information including haplogroups and archaeological features such as skeletal age-at-death (Mittnik *et al.* 2019, Schroeder *et al.* 2019, Fowler *et al.* 2022, Blöcher *et al.* 2023, Chyleński *et al.* 2023, Rivollat *et al.* 2023, Gnecchi-Ruscone *et al.* 2024, Penske *et al.* 2024, Rodríguez-Varela *et al.* 2024, Seersholm *et al.* 2024, Szécsényi-Nagy *et al.* 2025, Wang *et al.* 2025a, 2025b, Yüncü *et al.* 2025). These ancient pedigrees have yielded numerous insights into burial practices, kinship-based social structure, and the spread of disease throughout (pre)history.

Although the reconstruction of ancient pedigrees has furthered our understanding of the past, the manual pedigree reconstruction process remains an extremely time-consuming endeavor. Here, we introduce repare, an open-source software method that automates pedigree reconstruction using information readily available from aDNA data. repare iteratively builds up a set of candidate pedigrees from inferred pairwise degree-level kinship relations and uses optional supporting information, including haplogroups, runs of homozygosity (ROH), skeletal age-at-death estimates, and manual relation constraints to prune invalid pedigrees. Since kinship inference from aDNA is imperfect, especially for lower-coverage samples, repare explicitly considers alternative possibilities for kinship relations when building pedigrees (see Section 2.3). Using repare, we reconstruct pedigrees from a variety of simulated and published aDNA datasets. We find that repare can accurately reconstruct both simulated and published pedigrees from imperfect kinship relations, offering a valuable tool for automatic pedigree reconstruction.

## 2 Materials and methods

### 2.1 Iterative pedigree reconstruction

**Figure 1:**
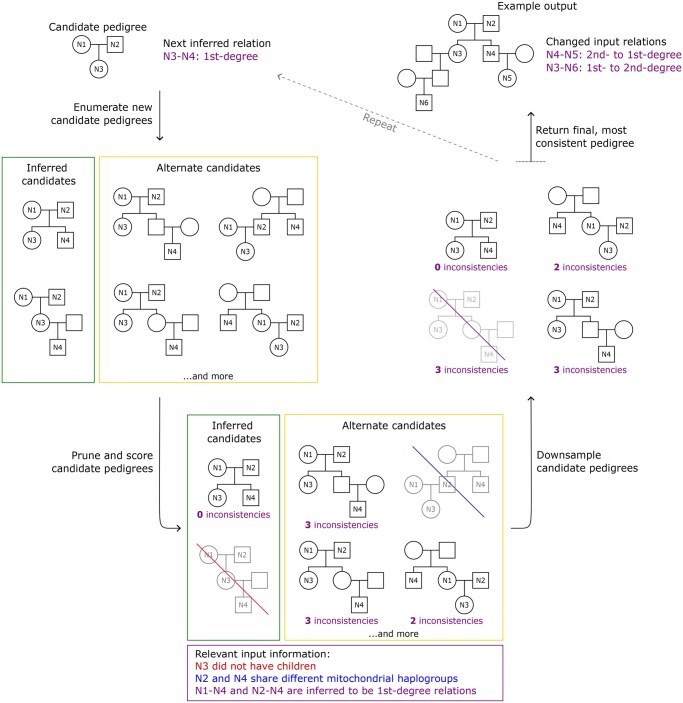
Diagram of repare’s pedigree reconstruction algorithm (see Section 2.1). At each algorithm iteration, an inferred kinship relation is incorporated into the current set of candidate pedigrees; alternative possibilities for the inferred relation are explicitly considered. The new set of candidate pedigrees is then pruned and downsampled before the next algorithm iteration.

repare’s pedigree reconstruction algorithm iteratively incorporates pairwise kinship relations into a set of progressively expanding candidate pedigrees (Fig. 1). Input kinship relations are sorted by ascending degree. Within each degree, kinship relations are sorted by descending individual connectivity so that relations involving individuals with more total relations are reconstructed first. We hypothesize that this ordering can help constrain later kinship relations by allowing the algorithm to first construct dense subpedigrees. At each algorithm iteration, repare considers a new degree-level kinship relation between two individuals. Inferred first- and second-degree relations are explicitly incorporated while considering potential alternative inferences (see Section 2.3); third-degree relation information is used to help score pedigrees (see Section 2.4).

Then, in each existing candidate pedigree, repare incorporates all possible exact kinship relations between the two individuals, barring relation type constraints (see Section 2.3). Because each degree-level relationship can correspond to multiple exact relations, repare duplicates candidate pedigrees before incorporating new relations in order to represent each possible scenario. In addition, repare uses placeholder individuals to represent unsampled individuals in candidate pedigrees. For each input relation, users can also set constraints on the exact relation type, e.g for first-degree relations where kinship relation inference methods can differentiate parental and full-sibling relations. repare does not restrict the number of kinship relations shared between individuals, enabling reconstruction of inbred relationships.

### 2.2 Pedigree pruning

Because degree-level kinship relations can be represented as multiple different relation configurations, the number of possible pedigrees can increase exponentially with the number of algorithm iterations (i.e. the number of input kinship relations). At each algorithm iteration, supplementary individual-level genetic and archaeological data are used to prune the set of candidate pedigrees. All such data are optional inputs, except for genetic sex, which we use to assign parentage. These supplementary data can include haplogroups and skeletal age-at-death estimates, which we use to remove candidate pedigrees with invalid relations. When available, ROH data are also used to eliminate candidate pedigrees where an individual appears inbred in the pedigree but is inferred to be non-inbred from ROH data. Finally, years-before-present data are used to eliminate candidate pedigrees with relations that are temporally improbable, e.g based on archaeological layer information or significant carbon dating differences.

### 2.3 Robustness to kinship inference errors

Inference of kinship relations from aDNA data is not perfectly accurate, and performance tends to deteriorate with lower sequence coverage and more distant kinship (Lefeuvre *et al.* 2025). As such, most datasets of inferred kinship relations from aDNA contain multiple errors, in which case a pedigree reconstruction algorithm that treats input kinship relations as fixed cannot reconstruct the true pedigree. In addition, in many cases, as few as one incorrect kinship relation can make finding even one valid pedigree impossible. To address this limitation, we implement an “inconsistency” scoring system for pedigrees: repare explicitly considers alternative possibilities for each input kinship relation; then, in each resulting candidate pedigree, each conflict between a relation in a candidate pedigree and the input kinship data is considered one inconsistency. For example, repare will try changing an inferred first-degree relation to a second-degree relation if that better fits the reconstructed pedigree. Then, after all input relations are incorporated, the pedigree with the fewest inconsistencies against the input data is returned.

### 2.4 Third-degree kinship relations

repare explicitly incorporates first- and second-degree kinship relations into candidate pedigrees and uses third-degree kinship relations as a tiebreaker mechanism to differentiate between otherwise equally plausible pedigrees. We choose not to explicitly incorporate third-degree kinship relations because of their relatively higher inference error rates and the additional complexity involved with enumerating all possible third-degree exact kinship relation types; not only are third-degree relations more common in most pedigrees, there are also more possible exact types of each third-degree relation. As such, we utilize the information from inferred third-degree kinship relations by introducing a separate “third-degree inconsistency” system that counts the number of extraneous third-degree kinship relations present in a candidate pedigree but not in the input kinship data. Since we do not explicitly incorporate third-degree kinship relations, we do not penalize third-degree relations present in the input data but not in a candidate pedigree. We use the third-degree inconsistency metric to differentiate candidate pedigrees with the same number of (first- and second-degree) inconsistencies.

### 2.5 Pedigree sampling

Even with pedigree pruning, we find that the number of candidate pedigrees grows far too quickly to enumerate every possible final pedigree. Therefore, we implement a sampling scheme to constrain the number of candidate pedigrees kept after each iteration. After incorporating each kinship relation into the set of candidate pedigrees, repare downsamples the candidate pedigree set to a fixed, user-defined number of pedigrees; we set the default value of this parameter to 1000. We downsample candidate pedigrees using a form of epsilon-greedy sampling (Sutton and Barto 2020) adapted for taking multiple actions. Standard epsilon-greedy sampling involves taking the greedy action (i.e. the action with the highest immediate reward) with probability 1−ε and taking a random action with probability ε. We adapt this approach for taking multiple actions as follows. Let *n* be the maximum number of candidate pedigrees to keep after each algorithm iteration and let ε be the “batch epsilon-greedy” parameter. Here, *n* can be viewed as the number of actions to take. To avoid running conventional epsilon-greedy sampling *n* times (without replacement), after each algorithm iteration we simply define the new, downsampled candidate pedigree set in two parts: ignoring integer rounding, we deterministically select the (1−ε)n candidate pedigrees with the fewest inconsistencies, and then uniformly sample n−(1−ε)n=εn pedigrees from the remaining set of candidate pedigrees. We set the default value of ε to 0.2, although we note that varying ε appears to have little effect on reconstruction performance (see Section 3.1). We evaluate the runtime and memory usage of repare in Note 3: Algorithm runtime and memory usage, available as supplementary data at *Bioinformatics* online.

### 2.6 Pedigree reconstruction evaluation

To measure pedigree reconstruction performance, we record two metrics: relation F1 score and degree F1 score. We define the relation F1 score of a reconstructed pedigree as the harmonic mean of precision and recall against the set of exact kinship relations (e.g. maternal grandparent-grandchild) in the ground-truth pedigree. Similarly, we define the degree F1 score of a reconstructed pedigree as the harmonic mean of precision and recall against the set of degree-level kinship relations (e.g. second-degree) in the ground-truth pedigree. Both metrics consider only first- and second-degree relations between sampled (non-missing) individuals. While the relation F1 score evaluates reconstruction of the exact simulated pedigree, we hypothesize that many ancient pedigree reconstruction problems are underdetermined such that multiple final pedigrees could plausibly agree with the input kinship relations data. Therefore, we also record degree F1 scores, which helps evaluate a more general structure of a predicted pedigree.

## 3 Results

### 3.1 Simulated pedigree reconstruction

**Figure 2:**
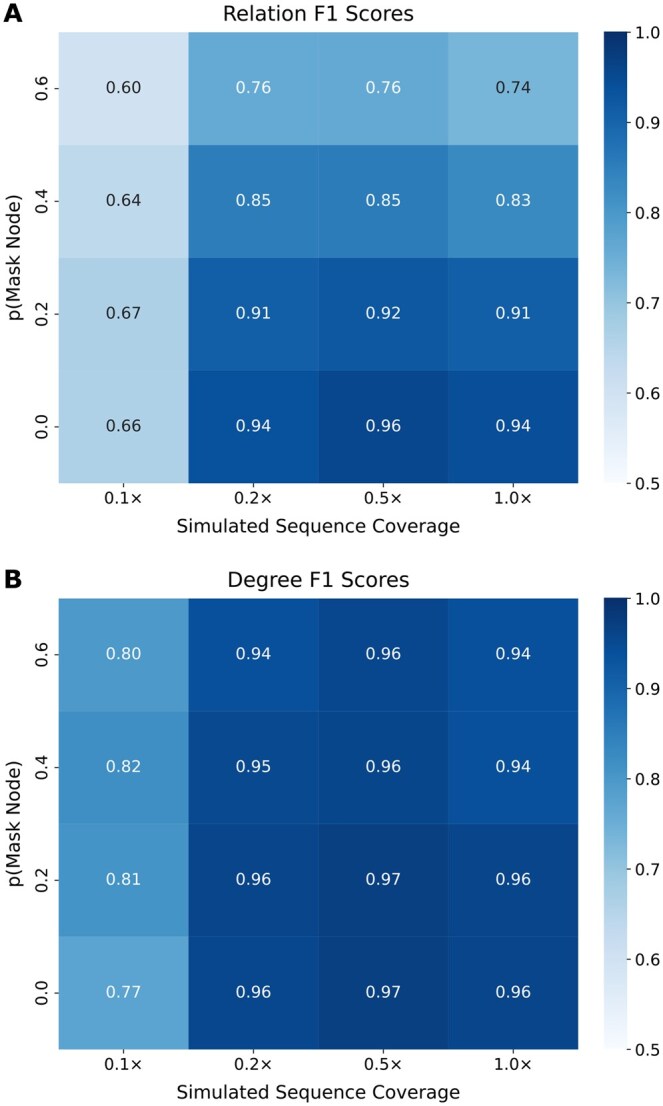
Pedigree reconstruction performance of repare on simulated data. We report reconstruction performance against ground-truth pedigrees using F1 scores, calculated over (A) exact kinship relations and (B) degree-level kinship relations (see Section 2.6).

We assess repare’s performance in reconstructing a variety of simulated pedigrees. To do so, we implement a custom pedigree simulator that generates ground-truth pedigrees and their accompanying genetic and age-at-death data, approximating the data collected from aDNA (see Note 1: Ancient pedigree simulation, available as supplementary data at *Bioinformatics* online). To evaluate repare’s performance under a range of data quality regimes, we vary the simulator’s p(mask node) parameter, which corresponds to pedigree missingness. We also vary its simulated sequence coverage parameter, which approximates kinship relation inference error rates based on those of KIN (Popli *et al.* 2023), a popular ancient kinship inference method, in a performance benchmark (Lefeuvre *et al.* 2025) (Fig. 2). For each configuration of the p(mask node) and simulated sequence coverage parameters, we use the same 100 simulated ground-truth pedigrees for consistency. Then, we mask and corrupt the data of each pedigree corresponding to the experiment’s parameters before providing it as input to repare. In this experiment, we reconstruct pedigrees using the default parameters of 1000 for the maximum number of candidate pedigrees kept after each algorithm iteration and 0.2 for the “batch epsilon-greedy” parameter (see Section 2.5).

We measure reconstruction performance with relation F1 and degree F1 scores (see Section 2.6). In terms of both metrics, repare accurately reconstructs pedigrees, with relation F1 scores exceeding 0.9 and degree F1 scores exceeding 0.95 when data quality is high; in addition, repare’s degree F1 performance is more robust to decreases in data quality. We observe a steep decline in performance as simulated sequence coverage drops from 0.2× to 0.1×. We note that repare appears to perform worse at 1.0× simulated sequence coverage than at 0.5× simulated sequence coverage. A likely explanation for this phenomenon is that, in the benchmark experiment used to determine kinship error rates (Lefeuvre *et al.* 2025), KIN performs worse in some respects at 1.0× simulated sequence coverage than at 0.5× simulated sequence coverage. We further analyze pedigree characteristics and pedigree-level performance in Note 2: Analysis of simulated pedigree reconstruction, available as supplementary data at *Bioinformatics* online. Notably, we find that reconstruction performance appears relatively robust to increases in ground-truth pedigree size at 0.5× simulated sequence coverage and less robust at 0.1× simulated sequence coverage. At both 0.5× and 0.1× simulated sequence coverage, we also observe a potential slight negative relationship between inbreeding proportion and reconstruction performance.

**Figure 3:**
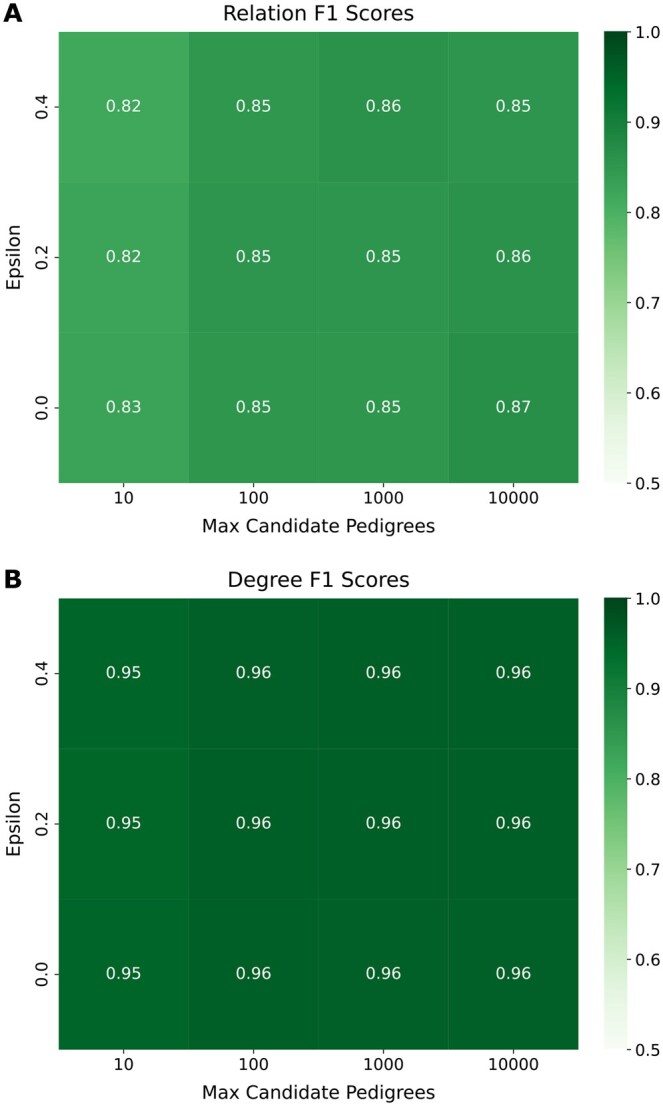
The effect of sampling parameters on repare’s pedigree reconstruction performance. We report reconstruction performance against ground-truth simulated pedigrees using F1 scores, calculated over (A) exact kinship relations and (B) degree-level kinship relations (see Section 2.6).

We also perform an experiment to quantify the effect of sampling parameters on reconstruction performance. Using the same 100 simulated ground-truth pedigrees as before, we evaluate repare’s performance while varying ε, the “batch epsilon-greedy” parameter and the maximum number of candidate pedigrees kept after each algorithm iteration (see Section 2.5) (Fig. 3). To generate data from the 100 simulated pedigrees, we fix p(mask node) to 0.4 and the simulated coverage level to 0.5×. We find that varying ε appears to have a minimal impact on reconstruction performance. On the other hand, increasing the maximum number of candidate pedigrees kept after each algorithm iteration appears to slightly improve performance. To balance performance and runtime considerations, we set the default value of this “max candidate pedigrees” parameter to 1000.

### 3.2 Published pedigree reconstruction

We also evaluate repare’s ability to reconstruct previously published ancient pedigrees. For each pedigree, we compile published kinship relation inference data as well as genetic and archaeological data, including haplogroups, genetic sexes, skeletal age-at-death estimates, and ROH. We provide these data as input to repare and assess its pedigree reconstruction performance using the published pedigree as ground truth. In certain cases, we also incorporate additional contextual information from case-specific inferences made by the authors of the published papers; this is to illustrate repare’s potential as an assistive tool in “human-in-the-loop” pedigree reconstructions. We include only author inferences that independently distinguish between possible kinship relations and exclude those that use information *post hoc* from adjacent kinship relations. For example, if authors modify an inferred kinship relation to “fit” better with the adjacent individuals in a pedigree, we do not include this inference. We evaluate repare’s reconstructed pedigrees against the corresponding published pedigrees using relation F1 and degree F1 scores (see Section 2.6). Some published pedigrees contain uncertain degree-level kinship relations where the authors do not infer an exact relation; these relations are often denoted with dotted lines in pedigree figures. Since repare only outputs pedigrees with exact relations, to calculate F1 scores over uncertain published relations, we consider a repare-reconstructed relation correct if it agrees with the degree of an uncertain relation and incorrect otherwise. Results for reconstructed pedigrees are summarized in Table 1.

**Table 1:** Results of repare’s reconstruction of published ancient pedigrees from their accompanying genetic and archaeological datasets.^a^

Pedigree site	Relation F1 score	Degree F1 score	Sampled	Kinship	Published
	(without → with	(without → with	Individuals	Inference	Pedigree
	human inferences)	human inferences)	in Pedigree	Errors	Missingness
Hazleton North (Fowler *et al.* 2022)	0.77 → 1.0	1.0 → 1.0	27	1	0.36
Nepluyevsky (Blöcher *et al.* 2023)	0.93 → 1.0	1.0 → 1.0	23^b^	4	0.38
Koszyce (Schroeder *et al.* 2019, Lefeuvre *et al.* 2024)	1.0 → N/A	1.0 → N/A	7	0	0.22
Gurgy “les Noisats” (Rivollat *et al.* 2023)	0.86 → N/A	0.95 → N/A	82	14	0.41

a All pedigrees were reconstructed with default sampling parameters (see Section 2.5). We report relation F1 and degree F1 scores (see Section 2.6) to evaluate performance. When applicable, we report performance before and after providing repare with additional information from author inferences used to reconstruct the published pedigree. To provide context on reconstruction difficulty, we report the number of sampled individuals related to at least one other individual in the published pedigrees, the number of first- and second-degree kinship inference errors in the reported data with respect to the published pedigrees, and the missingness of each published pedigree. Published pedigree missingness is calculated by counting the number of plotted placeholder individuals, without considering uncertain relations, and dividing by the total number of individuals related to at least one other individual.

b The Nepluyevsky pedigree contains 23 individuals, but it is inferred by KIN to include a pair of identical twins, which repare treats as one individual.

### 3.3 Hazleton North site

We first assess repare’s performance in reconstructing a Neolithic pedigree from the Hazleton North site (Fowler *et al.* 2022), using published genetic and archaeological data. The published pedigree was manually reconstructed using pairwise kinship relations and additional genetic and archaeological information, including haplogroups, ROH, and skeletal age-at-death estimates. Degree-level kinship relations were inferred using relatedness coefficients (*r*), and first-degree relation types (parental or sibling) were inferred using IBD information. Using only this published data as input, repare’s reconstructed pedigree achieves a relation F1 score of 0.77 and a degree F1 score of 1.0 against the published pedigree. However, Fowler *et al.* (2022) incorporated additional logical inferences to reconstruct their final published pedigree. In some cases, the directionality of parent-offspring kinship relations was determined through haplogroup rarity; e.g. if a genetic male and a genetic female share a rare mitochondrial haplogroup as well as a parent-offspring kinship relation of unknown directionality, it is more likely that the female is the mother of the male and directly passed that haplogroup down. In another case, the final pedigree was selected over an alternative pedigree after manual inspection of the location of recombination breakpoints. When we include this additional information in repare’s inputs as kinship relation constraints, the reconstructed pedigree achieves perfect relation F1 and degree F1 scores of 1.0. We note that the reconstructed pedigree does not include SP4m as a connected individual, since SP4m’s closest inferred kinship relation is of the third degree. Overall, this result demonstrates repare’s effectiveness not only as a fully automatic pedigree reconstruction tool but also as a useful tool for iterative semi-automatic analyses.

### 3.4 Nepluyevsky site

**Figure 4:**
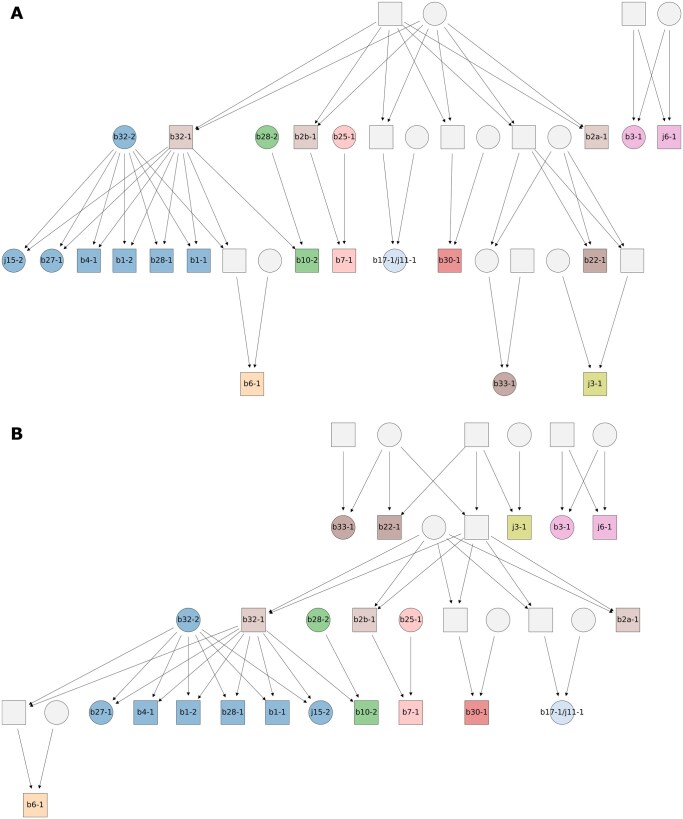
(A) The published Nepluyevsky pedigree (Blöcher *et al*. 2023) and (B) the original repare-reconstructed Nepluyevsky pedigree. Gray nodes without an ID label correspond to placeholder individuals. Nodes corresponding to non-placeholder individuals are colored by the individuals’ mitochondrial haplogroups. Unrelated individuals are not included in these plots.

We also evaluate repare’s reconstruction of a Bronze Age pedigree from the Nepluyevsky site (Blöcher *et al.* 2023). Similar to the Hazleton North pedigree, the published Nepluyevsky pedigree was manually reconstructed using pairwise kinship relations and supporting information, including haplogroups and skeletal age-at-death estimates. Degree-level kinship relations and first-degree relation types were inferred using KIN (Popli *et al.* 2023). Using this published data as input, repare’s reconstructed pedigree achieves a relation F1 score of 0.93 and a degree F1 score of 1.0 against the published pedigree. However, we note that repare’s reconstructed pedigree appears to represent a valid alternative pedigree for the data. There are a number of second-degree relations for which repare inferred a different relation type than in the published pedigree. For these relations, it is unclear how the final relation type was determined for the published pedigree. Therefore, we believe that repare’s reconstructed pedigree represents a plausible alternative pedigree given the available data. The published pedigree and original reconstructed pedigree are shown in Fig. 4. Assuming each differing relation type was determined through author inferences, we encode this information into repare’s inputs as relation constraints, similar to our procedure for the Hazleton North pedigree (see Section 3.3). When we do so, the reconstructed pedigree exactly matches the published pedigree.

**Figure 5:**
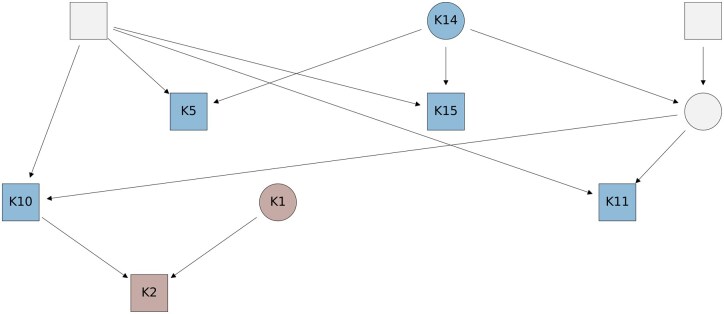
The repare-reconstructed Koszyce pedigree. Gray nodes without an ID label correspond to placeholder individuals. Nodes corresponding to non-placeholder individuals are colored by the individuals’ mitochondrial haplogroups.

### 3.5 Koszyce site

To evaluate performance on a dataset including complex kinship relations, we use repare to reconstruct a Late Neolithic (sub)pedigree from the Koszyce site (Schroeder *et al.* 2019). This site includes a pedigree inferred to contain complex kinship relations, including two pairs of individuals related at both the second and third degrees. This seven-individual pedigree, involving a subset of the Koszyce site’s related individuals, was reconstructed in a later analysis of the individuals’ kinship relations (Lefeuvre *et al.* 2024). As such, we primarily use the kinship relations inferred by GRUPS-rs in this later analysis (Lefeuvre *et al.* 2024). Since GRUPS-rs does not infer first-degree relation types, we use inferred first-degree relation types from the original article describing the Koszyce site (Schroeder *et al.* 2019). We also obtain individual-level metadata, including haplogroups and skeletal age-at-death estimates, from the original article. Given this input information, repare successfully reconstructs the seven-individual pedigree (Fig. 5). Notably, GRUPS-rs infers that K14 shares a first-degree relation with the mother of K10 and K11; it does not specify the exact relation type. repare successfully recovers this first-degree relation.

### 3.6 Gurgy “les Noisats” site

**Figure 6:**
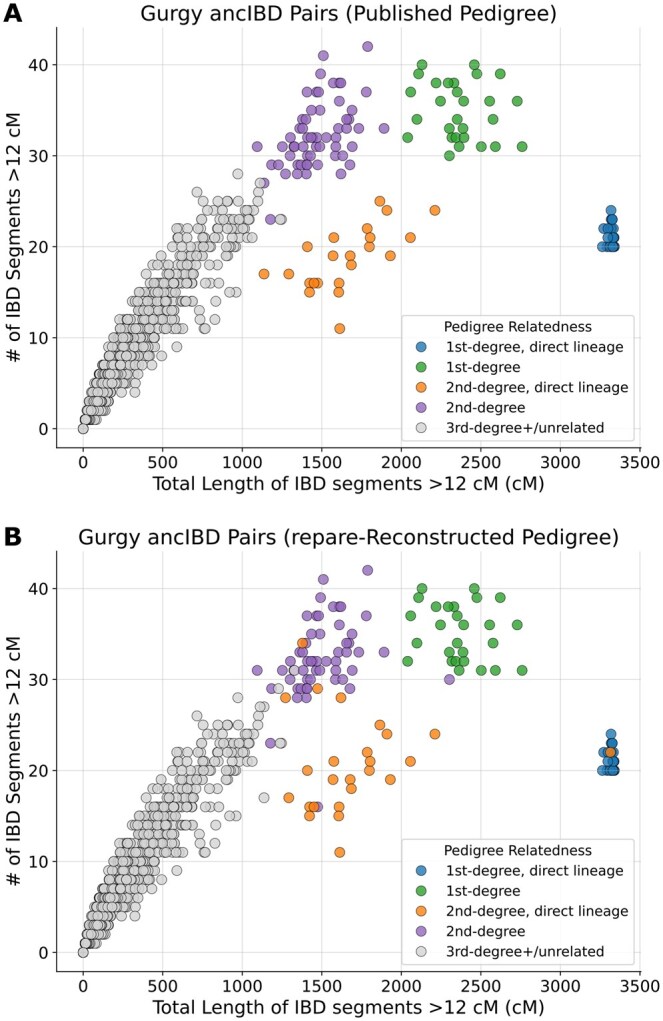
Inferred Gurgy IBD data for pairs of individuals, colored by kinship relation classifications in (A) the published Gurgy pedigree (Rivollat *et al*. 2023) and (B) the repare-reconstructed Gurgy pedigree.

Finally, we assess repare’s reconstruction of a Neolithic pedigree from the Gurgy ‘les Noisats’ site (Rivollat *et al.* 2023). Similar to the previous pedigrees, the published Gurgy pedigree was manually reconstructed using pairwise kinship relations and supporting information, including haplogroups and skeletal age-at-death estimates. For the data used to reconstruct the published pedigree, degree-level kinship relations were inferred using READ (Kuhn *et al.* 2018), and first-degree relation types were inferred using lcMLkin (Lipatov *et al.* 2015). In addition, uncertain relations were investigated using BREADR (Rohrlach *et al.* 2025). These data sources had many mutual conflicts, which were manually resolved by the pedigree authors. Therefore, instead of attempting to unify the multiple kinship data sources for input to repare, we evaluate reconstruction of the Gurgy pedigree using later-reported kinship data from READv2 (Alaçamlı *et al.* 2024), a kinship inference method that infers both degree-level relations and first-degree relation types. With this input data, repare achieves a relation F1 score of 0.86 and a degree F1 score of 0.95 against the published pedigree. This is the only published pedigree in this work for which repare cannot achieve perfect relation F1 and degree F1 scores using published input information. The published Gurgy pedigree was validated using inferred IBD data from ancIBD (Ringbauer *et al.* 2024), which offers a relatively independent measurement of a pedigree’s plausibility. To compare the plausibility of the published pedigree and the repare-reconstructed pedigree, we plot these published IBD data (Rivollat *et al.* 2023), color-coding pairs of individuals by their pedigree relatedness (Fig. 6). The published pedigree is noticeably more concordant with the IBD data, as its pedigree-relatedness classifications correspond more strongly with the IBD-relatedness “clusters.” We discuss a possible reason for repare’s shortcoming in reconstructing the Gurgy pedigree, as well as directions for future work, in Section 4.

## 4 Discussion

We evaluate repare on a variety of simulated and published ancient pedigrees and find that it achieves strong reconstruction results under data quality conditions typical of aDNA. In addition, through reconstruction of published, manually reconstructed pedigrees, we highlight repare’s ability to flexibly integrate genetic and archaeological constraints alongside pairwise kinship relations (see Section 3.2). In repare’s reconstructions of the Hazleton North and Nepluyevsky pedigrees, explicit incorporation of site-specific archaeological and genetic context, such as haplogroup rarities and recombination breakpoints, improved reconstruction to the point of successfully recovering the published pedigrees up to their first- and second-degree relations. repare is also able to reconstruct pedigrees involving complex kinship relations, as we observe for the Koszyce seven-individual pedigree.

We find that repare was unable to exactly recover the published Gurgy “les Noisats” pedigree. Compared to the published Gurgy pedigree, the repare-reconstructed Gurgy pedigree contains the same number of first- and second-degree inconsistencies and fewer third-degree inconsistencies, computed against the READv2-inferred kinship relations (see Sections 2.3 and 2.4). However, the repare-reconstructed Gurgy pedigree does not appear to be more plausible than the published Gurgy pedigree (Fig. 6). In other words, although repare successfully reconstructs *most* of the Gurgy pedigree, its specific modifications to the input kinship relations appear to produce a pedigree that is overall less plausible than the published pedigree. As such, we believe this pedigree highlights a limitation of repare’s discrete inconsistency metric. repare treats all first- and second-degree inconsistencies as equivalent and all third-degree inconsistencies as equivalent. However, it is hypothetically possible to determine relative likelihoods of inconsistencies and assign more fine-grained scores to each inconsistency, e.g. using IBD data. It may also be possible to jointly infer likelihoods of entire pedigrees, bypassing the need to score individual inconsistencies. Ultimately, we believe the development of a continuous inconsistency scoring system, potentially leveraging additional sources of relatedness information such as IBD data, presents an opportunity for improvement in future versions of repare.

As seen in repare’s performance on simulated pedigrees (see Section 3.1), pedigree reconstruction is a process sensitive to input data quality. The exponential nature of iterative pedigree reconstruction makes it difficult to correct an excessive number of kinship inference errors. However, in sufficiently dense pedigrees, information from multiple adjacent kinship relations can help resolve pedigree topologies. In addition, it is still possible to make inferences about social dynamics and other general questions from pedigrees with some error. In this sense, reconstructing degree-level relations for an approximate pedigree topology can be nearly as productive as reconstructing exact relation types, which is a more difficult task.

To our knowledge, repare is the first pedigree reconstruction method to primarily utilize pairwise kinship relations. This makes repare particularly suitable for the reconstruction of ancient pedigrees. Existing ancient pedigrees reported in the literature are largely manually reconstructed, highlighting a need for automatic reconstruction methods that tolerate the relatively poor data quality of aDNA. We show that repare represents a powerful method for the automatic reconstruction of ancient pedigrees using only data commonly collected in aDNA analyses. In addition, repare can flexibly integrate user-inferred pedigree constraints, allowing users to guide reconstruction analyses through an iterative “human-in-the-loop” process.

## Supplementary Material

btag251_Supplementary_Data

## Data Availability

No new genomic data were generated in this work. Data used to reconstruct published ancient pedigrees are available from Schroeder *et al.* (2019), Fowler *et al.* (2022), Blöcher *et al.* (2023), Rivollat *et al.* (2023), Alaçamlı *et al.* 2024, Lefeuvre *et al.* 2025. Data used to reconstruct simulated ancient pedigrees are available on Zenodo at https://doi.org/10.5281/zenodo.19716772.
